# IL-35, IL-37, and IL-38 in acute pancreatitis: proposed immunopathogenic mechanisms and therapeutic potential

**DOI:** 10.3389/fimmu.2026.1728737

**Published:** 2026-02-09

**Authors:** Hailong Yan, Xingbo Dang, Gongliang Du, Longyang Ma, Wei Dai, Shisan Bao, Xiujuan Huang

**Affiliations:** 1 Department of Emergency Surgery, Shaanxi Provincial People’s Hospital, Xi’an, Shaanxi, China; 2 Center for Evidence-based Medicine, Gansu University of Chinese Medicine, Lanzhou, Gansu, China; 3 Department of Hematology, Shaanxi Provincial People’s Hospital, Xi’an, Shaanxi, China

**Keywords:** acute pancreatitis, IL-35, IL-37, potential application, protective

## Abstract

Acute pancreatitis (AP) is driven by premature enzyme activation, pancreatic tissue injury, and dysregulated immune responses. IL-35 and IL-37 are key anti-inflammatory mediators whose dynamic regulation influences disease severity. Circulating IL-35 is typically upregulated in AP, functioning through STAT1/STAT4 signalling to suppress effector T-cell proliferation, inhibit Th1/Th17 differentiation, and promote expansion of regulatory T and B cells, thereby limiting pro-inflammatory cytokine release (e.g., TNF, IL-6, IL-17) and reducing local and systemic inflammation. In contrast, IL-37 is often downregulated early in AP, impairing its ability to suppress NF-κB and MAPK signalling, restrain dendritic cell and macrophage activation, and reduce gasdermin D (GSDMD)-mediated pyroptosis. Experimental restoration of IL-37 diminishes neutrophil and macrophage infiltration, mitigates pancreatic necrosis, and modulates STAT signalling. The interplay of upregulated IL-35 with insufficient IL-37, within a broader cytokine network, emphasises a compensatory yet incomplete anti-inflammatory response. IL-38, although mechanistically promising, has not yet been characterised in human or animal AP models, and its clinical translation remains hypothetical. These insights suggest that clinical strategies—such as recombinant IL-37 therapy, IL-35 modulators, or combination cytokine-targeted interventions—may restore immune homeostasis and improve outcomes in AP.

## Introduction

Acute pancreatitis (AP) is an escalating global health burden and a leading cause of gastrointestinal hospitalisation (1), with an incidence of 13–45 per 100,000 annually. Gallstones, alcohol consumption, hypertriglyceridaemia, and certain medications are common triggers, while genetic and autoimmune factors contribute in specific populations (2). Chronic pancreatitis, though less frequent, often arises from long-term alcohol use, hereditary factors, or autoimmune conditions. Given marked regional and demographic variations, identifying population-specific risk factors is essential for effective prevention and management strategies. Despite advances in supportive care, AP continues to cause substantial mortality, morbidity, and healthcare costs (1, 2), highlighting the urgent need for novel therapeutic strategies, particularly those addressing the lack of immune-targeted treatments and the persistently high mortality associated with severe AP.

AP typically presents with sudden-onset severe epigastric pain radiating to the back and elevated pancreatic enzymes, with imaging modalities such as ultrasound or CT guiding diagnosis and severity assessment. Chronic pancreatitis develops more insidiously, with recurrent abdominal pain, malabsorption, and progressive endocrine insufficiency. Early recognition of severity and complications is critical for improving outcomes. Current management remains largely supportive, including fluid resuscitation, analgesia, and early nutritional support, while severe cases may require intensive care and interventions for complications (3). Experimental therapies targeting immune modulation and cytokine regulation are under active investigation to limit pancreatic injury and disease progression (4). Given this, cytokine-based immune modulation - particularly involving IL-35 and IL-37 - has emerged as a promising area of translational research.

Multiple triggers of AP (1) damage pancreatic acinar cells, causing trypsinogen leakage, premature enzyme activation, and initiation of the inflammatory cascade (2). Beyond the initial enzymatic injury, a dysregulated immune response amplifies tissue damage and provokes local as well as systemic inflammation, determining disease severity from mild oedematous pancreatitis to necrotising forms with multi-organ failure (2). Subsequently, disturbed host immune homeostasis further amplifies this injury process, with cytokines orchestrating whether inflammation resolves or escalates (5). Modulating cytokine activity – either by suppressing pro-inflammatory mediators or enhancing anti-inflammatory cytokines -significantly dictate outcomes, likely by damaging or protecting acinar cells, ducts, and infiltrating leucocytes (6). The most Recent Nobel-recognised discoveries in immune tolerance have underscored how peripheral immune regulation governs tissue-specific inflammatory diseases, offering some insight into AP pathogenesis (7). This breakthrough has substantially deepened our understanding of the mechanisms underlying the development of AP in susceptible individuals, particularly through dysregulation of the balance between pro- and anti-inflammatory responses. These seminal findings provide a stronger scientific foundation for improving the management of AP. Among cytokines maintaining this balance, IL-35 and IL-37 have emerged as key anti-inflammatory mediators with therapeutic promise in AP.

## IL-35: biology and mechanistic function in AP

IL-35, a member of the IL-12 cytokine family, functions primarily as an anti-inflammatory regulator by promoting regulatory T (Treg) cells and inhibiting excessive immune responses (8). It is produced mainly by Treg cells, Breg cells, and other immune cells in response to inflammatory stimuli (9). Mechanistically, IL-35 suppresses effector T-cell proliferation, inhibits Th1 and Th17 differentiation, and promotes the expansion of Treg and Breg populations (10). In various inflammatory conditions, IL-35 exerts protective effects: it promotes M2 macrophage polarisation in psoriasis (11), reduces pathological responses in rheumatoid arthritis (12), and confers cardiovascular protection (13). IL-35 signals primarily through STAT1 and STAT4, repressing transcription of pro-inflammatory cytokines such as TNF, IL-6, and IL-17 (14), thereby limiting immune cell activation and protecting tissues.

IL-35 may mitigate pancreatic injury and modulate inflammation in patients with AP, owing to its potent anti-inflammatory activity during the acute phase of the disease. Clinical studies show circulating IL-35 is elevated in patients with AP compared with matched controls, independent of sex, age, smoking status, hypertension, hyperlipidaemia, or BMI (15). Elevated IL-35 correlates with disease severity, persisting throughout the first week, and is independent of aetiology (alcoholic, gallstone-associated, or idiopathic AP) (15). These findings suggest that IL-35 elevation reflects disease progression rather than specific triggers. Based on its anti-inflammatory properties (8, 16), IL-35 likely originates from recruited leucocytes and/or injured acinar cells as a compensatory response to inflammatory stress (15). However, such mechanisms appear insufficient to fully counteract overwhelming immune activation, such as under cytokine storms, and the precise molecular pathways remain incompletely defined.

Autoimmune pancreatitis (AIP) is a distinct immune-mediated fibro-inflammatory pancreatic disease characterised by dysregulated immune responses (2, 17). Based on the International Consensus Diagnostic Criteria, AIP is classified into two subtypes: Type I IgG4-related autoimmune pancreatitis, also termed lymphoplasmacytic sclerosing pancreatitis (LPSP), and Type II idiopathic duct-centric pancreatitis (IDCP) (18). Type I AIP represents a pancreatic manifestation of systemic IgG4-related disease, predominantly affecting adults and more frequently observed in Asian populations, and is characterised by elevated serum IgG4 levels (>135 mg/dL), abundant IgG4-positive plasma cell infiltration, an IgG4/IgG ratio >0.4, dense lymphoplasmacytic inflammation, fibrosis, and obliterative phlebitis (19, 20). IgG4’s pathogenesis involves a systemic autoimmune response, likely triggered by various microbes in gastrointestinal system, leading to massive infiltration of IgG4 plasma cells, which secrete anti-inflammatory IgG4 and pro-fibrotic cytokines, causing fibrosis, inflammation, and organ damage, involving complex T-cell (Th2/Treg) responses and characteristic storiform fibrosis and obliterative phlebitis.

In contrast, Type II AIP typically occurs in younger patients, is more prevalent in European and North American populations, lacks systemic and serological IgG4 abnormalities, and is often associated with inflammatory bowel disease. Histopathologically, Type II AIP is characterised by predominant neutrophilic infiltration with granulocytic epithelial lesions, minimal IgG4-positive plasma cells, and duct-centred epithelial injury leading to pancreatic parenchymal destruction and ductal obstruction (20). Early and accurate differentiation between these subtypes is essential for precision management and prognostic assessment.

The role of IL-35 is further illustrated, showing circulating and tissue IL-35 levels are significantly elevated in Type 1 AIP and alcoholic pancreatitis at both mRNA and protein levels (21). IL-35 correlates with other proinflammatory cytokines such as IL-27, IL-28A, and IL-29, but not with conventional biomarkers (bilirubin, amylase, liver enzymes, WBC, CRP, HbA1c). Positive correlations with IL-29 and IgG4 (21) suggest that IL-35 restrains dysregulated immunity during cytokine storms, although its ability to restore homeostasis in patients with AIP remains limited in individuals with pre-existing extensive immune dysfunction (22).

Consistent with immune dysregulation in AIP, natural Tregs (nTregs) are reduced, while effector Tregs (eTregs) are increased (21). nTregs maintain baseline immune tolerance, and their reduction may predispose to AP initiation. Acinar stress and release of damage-associated molecular patterns (DAMPs) could trigger inappropriate trypsinogen activation, promoting inflammation in genetically or immunologically susceptible hosts. In contrast, expansion of eTregs likely represents a compensatory response to ongoing inflammation. IL-35 induces inducible IL-35–producing regulatory T cells (iTr35) (23), a distinct Foxp3^-^ regulatory T-cell subset that reinforces immunosuppression through autocrine and paracrine IL-35 signalling. A reduction in thymus-derived nTreg cells may contribute to disease initiation, whereas subsequent expansion of iTr35 cells, functionally within the eTreg compartment, appears to occur at later stages as an attempt to limit inflammatory progression, albeit insufficiently in susceptible individuals.

The positive correlation between IL-35 and IgG4 in Type 1 AIP provides mechanistic insight into the overlap between AIP and IgG4-related disease (IgG4-RD) (22). IL-35 promotes B-cell class switching to IgG4, contributing to fibrosing and chronic features of AIP. Histochemistry confirms that both Ebi3 and IL-12p35 subunits [which form IL-35 (8)] are more abundant in AIP than in alcoholic pancreatitis, reinforcing IL-35 upregulation as a compensatory response. These findings suggest IL-35 may serve as a biomarker distinguishing AIP from other forms of pancreatitis, and modulation of the IL-35–Treg–IgG4 axis could offer therapeutic potential.

Currently, the relationship between IL-35 and Type II AIP remains unexplored and warrants further investigation.

Animal models provide further mechanistic insight. Following AP onset, neutrophils and macrophages rapidly infiltrate the pancreas (24), mediated by CD18 (25). Ablation of CD18 or depletion of these cells reduces protease activation, amylase/lipase levels, and tissue damage. Anti-neutrophil serum decreases myeloperoxidase activity, and macrophage depletion reduces trypsinogen activation (24). Upregulated TNF amplifies local damage (26), enhancing pro-inflammatory responses in the affected pancreas. These findings underscore the interplay between pro-inflammatory innate immune responses and compensatory IL-35 upregulation; however, the animal model does not fully recapitulate AP or Type I/Type II AIP.

Furthermore, IL-35 also acts within a wider cytokine network, including IL-37, IL-36, and IL-38. IL-37 restrains innate immune activation (discussed below), IL-38 modulates immunity context-dependently, and IL-36 amplifies pro-inflammatory cascades (27). In AP, an imbalance between elevated IL-36 and insufficient IL-35, IL-37, and IL-38 may drive unchecked inflammation. Although IL-35 is upregulated as a compensatory response, this increase may be insufficient to restore immune balance, highlighting the need for therapies targeting multiple anti-inflammatory pathways during cytokine storms.

## IL-37: biology and mechanistic role in AP

IL-37, a member of the IL-1 family, exerts context-dependent immunomodulatory effects. Intracellularly, it translocates to the nucleus, binds Smad3, suppresses transcription of pro-inflammatory genes, inhibits NF-κB and MAPK signalling, and limits activation of dendritic cells and macrophages. Extracellularly, IL-37 signals via IL-18Rα and recruits SIGIRR/IL-1R8, exerting anti-inflammatory effects under mild conditions but potentially enhancing inflammation during severe tissue injury or high cytokine burden (28). Through these dual mechanisms, IL-37 calibrates immune responses according to the intensity and context of inflammatory stress.

In AP, circulating IL-37 levels are reduced compared with healthy controls and show dynamic temporal changes. The greatest reduction occurs 72–96 hours after onset, particularly in severe AP, followed by gradual recovery, suggesting an endogenous attempt to restore homeostasis (29). Lower IL-37 levels correlate with elevated systemic inflammatory markers (LDH, CRP, IL-6), BMI, and pancreatic necrosis. ROC analysis indicates IL-37 predicts necrosis with high sensitivity (90%) and specificity (79.4%), supporting its potential prognostic utility (29).

The cellular source of IL-37 remains uncertain but may include infiltrating leucocytes and possibly acinar cells. Identifying the source and/or correlation with severity of AP, particularly at the initial stage, determining the onset of AP would really revealing the pathogenesis of host immune peripheral tolerance/intolerance. However it is almost impossible and/or inappropriate to obtain some human biopsies at appropriate stages of the development of AP, however, these are very hard to impossible to obtain, although it might be possible to have some post-mortem individuals with fulminate AP in occasional cases, the use of post-mortem AP tissues, as applied in other diseases (30), due to ethical issue, may help clarify this. Dead tissue can be obtained but this only really represents end-stage disease in most cases (there may be the occasional death in a person who is debilitated then develops AP, e.g. someone with severe heart disease who can’t handle the stress).

Animal studies provide mechanistic insight: in caerulein-induced AP, IL-37 transgenic mice show reduced necrosis, lower amylase, lipase, and IL-1β, and decreased macrophage and neutrophil infiltration (29), supporting the protective role of IL-37 in the development of AP. Such finding is in line with the data from *in vitro* study, recombinant IL-37 suppresses cholecystokinin-induced cell death in both the 266–6 acinar cell line and primary pancreatic acinar cells (29).

IL-37 also influences macrophage polarisation. M1/M2 balance is a key determinant of inflammatory outcomes in AP (31), and IL-37 promotes M2 polarisation, limits inflammasome activation, and restrains systemic inflammation. Recombinant IL-37 reduces gasdermin D (GSDMD)-mediated pyroptosis and modulates STAT signalling, breaking the cycle of acinar injury, DAMP release, and immune activation. GSDMD-knockout mice fail to respond, confirming IL-37’s dependence on this pathway.

Therapeutically, IL-37 - either as recombinant protein or mimetics—represents a promising approach to modulate dysregulated immunity in AP. However, the timing, administration route, and dosing require careful optimisation to maximise benefit and avoid potential pro-inflammatory effects in severe disease. Similar protective effects have been observed in cancer (31, 32), CNS autoimmunity (33), and cardiovascular injury (34), supporting broad relevance. Future studies should dissect molecular pathways mediating IL-37’s effects to guide targeted interventions.

## IL-38: speculative role

IL-38 modulates immunity in a context-dependent manner, but data in AP are currently lacking. It is hypothesised that IL-38 may protect against inflammation-mediated injury synergistically with IL-35 and IL-37, warranting future investigation (35). Although mechanistic insight is limited, IL-38 could fine-tune both innate and adaptive immune responses, potentially complementing IL-37-mediated anti-inflammatory pathways.

## Clinical relevance and therapeutic potential

IL-35 and IL-37 have shown protective and regulatory roles in other diseases, including in breast cancer (32), hepatocellular carcinoma (36), and inflammatory bowel disease (37). In AP, IL-35-based strategies may provide consistent anti-inflammatory effects, whereas IL-37-based therapies require careful consideration of timing, localisation, and disease severity to maximise benefit while avoiding potential pro-inflammatory outcomes.

Understanding the interplay of IL-35, IL-37, and possibly IL-38 may inform biomarker development for predicting disease severity and guiding personalised interventions. IL-37, in particular, has prognostic potential due to its temporal changes correlating with necrosis and systemic inflammation. Targeting IL-37-mediated pathways may reduce tissue injury, modulate immune responses, and limit systemic complications.

## Future directions and integrative approaches

The precise sources and localisation of IL-35 and IL-37 remain incompletely defined. Identifying genes regulating their expression in pancreatic tissue, including polymorphisms or upstream signalling molecules, may provide critical insight. Integrative approaches combining graph neural networks with causal discovery represent promising strategies (38). Future studies could employ alternative causal algorithms, such as linear non-Gaussian acyclic models, and genome-wide CRISPR screens (39) to validate gene essentiality. Protein–protein interaction databases such as STRING and BioGRID may determine whether candidate genes are embedded in established regulatory modules or pathways (38).

**Figure 1:**
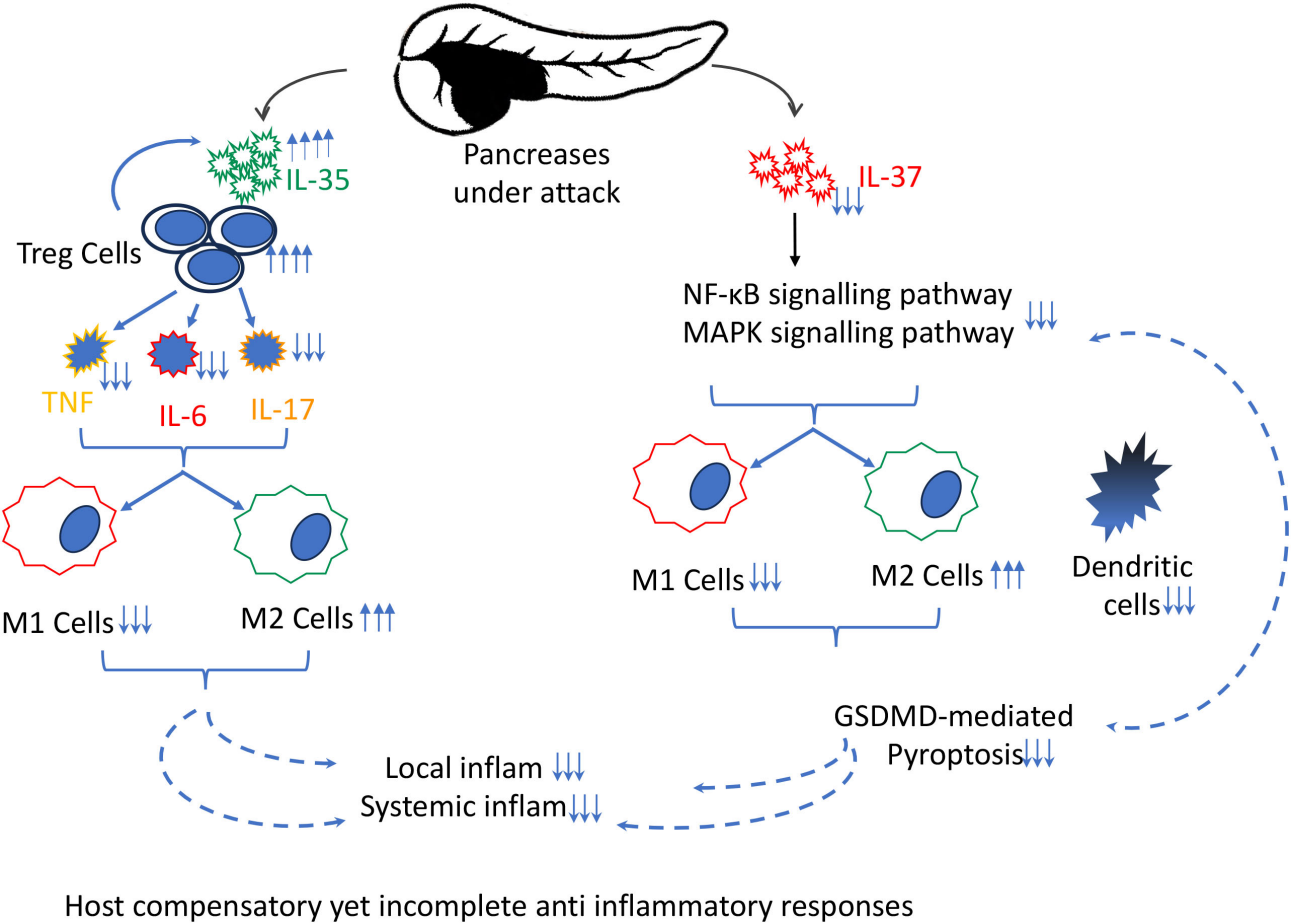
The schematic diagram illustrates the key immune-mediated processes that perpetuate acute pancreatitis (AP). In the affected pancreas, there is an imbalance between pro- and anti-inflammatory factors. The anti-inflammatory response is initiated by IL-35, which promotes the expansion and function of regulatory T (Treg) cells and modulates macrophage polarization (M1 to M2), working to suppress pro-inflammatory cytokines (e.g., IL-6, TNF, IL-17). Despite these host compensatory, yet incomplete, anti-inflammatory effects, excessive inflammation persists. Concurrently, a downregulation of IL-37 expression further contributes to the imbalance between M1 and M2 macrophages and reduces the dendritic cell population. These immune alterations are mechanistically mediated through the activation of NF-κB and MAPK signalling pathways, and involve GSDMD-driven pyroptosis, collectively perpetuating local and systemic inflammatory responses characteristic of acute pancreatitis.

Future studies should also employ multiplex immunohistochemistry (40) to characterise M1/M2 macrophage polarisation, assess interactions with neutrophils and other immune subsets, and monitor dynamic shifts under cytokine treatment. Mechanistically, IL-35 and IL-37 are expected to act *via* multiple pathways: inhibition of NF-κB and MAPK, suppression of STAT1/STAT3 transcription, restraint of NLRP3 inflammasome activation, and limitation of GSDMD-mediated pyroptosis. These interventions could reduce DAMP release and downstream cytokines, breaking the cycle of acinar injury, immune recruitment, and systemic inflammation. A schematic diagram illustrates the functional interactions of these cytokines and the associated pathways that drive the pathogenesis of AP (**Figure 1**).

## Limitations and safety considerations

Although exogenous IL-35 and/or IL-37 are promising therapeutic candidates, challenges remain, particularly regarding safety and systemic immunological effects. Recombinant IL-37 has shown efficacy in animal models, but comprehensive evaluation of pharmacokinetics, therapeutic efficacy, and systemic safety is still required for both cytokines. IL-38, although mechanistically promising, has not yet been characterised in human or animal AP models, and its clinical translation remains hypothetical.

## Conclusion

IL-35 and IL-37 play distinct yet interconnected roles in AP. IL-35 acts as a consistent anti-inflammatory regulator by enhancing Tregs and suppressing pro-inflammatory cytokines, while IL-37 exhibits dual roles: intracellularly suppressing inflammation and extracellularly exerting protective or context-dependent effects. The balance between these cytokines likely influences AP severity, complications, and recovery. Mechanistic insights into their regulation may guide development of cytokine-based therapies to modulate inflammation, limit pancreatic injury, and improve clinical outcomes in AP.
